# Evaluating the appropriateness and the factors associated with sodium-glucose co-transporter 2 inhibitors prescribing in a Middle Eastern country: a cross-sectional study

**DOI:** 10.1007/s11096-024-01828-5

**Published:** 2024-11-21

**Authors:** Nancy Zaghloul, Ahmed Awaisu, Ahmed Mahfouz, Zainab Ali, Sumaya Alyafei, Hazem Elewa

**Affiliations:** 1 College of Pharmacy, QU Health, Qatar University, P.O. Box 2713, Doha, Qatar; 2 Pharmacy Department, Heart Hospital, Hamad Medical Corporation, P.O. Box 3050, Doha, Qatar; 3 Biomedical and Pharmaceutical Research Unit, QU Health, Qatar University, P.O. Box 2713, Doha, Qatar

**Keywords:** Appropriateness of prescribing, Diabetes mellitus, Factors, Oral antidiabetic drugs, Sodium-glucose transporter 2 inhibitors

## Abstract

**Background:**

Sodium glucose co-transporter 2 inhibitors (SGLT2is) are a novel class of oral antidiabetic drugs (ADDs). Studies evaluating the appropriateness of SGLT2is prescribing, and the factors associated with their initiation in the Middle East region are lacking.

**Aim:**

This study aimed to evaluate the appropriateness of prescribing SGLT2is based on indication, dosing, and contraindication and determine the factors associated with their initial prescribing.

**Method:**

In this cross-sectional study, a cohort of 650 patients newly prescribed SGLT2is (n = 400) and/or any other oral ADDs (n = 250) during 2020 were included. Data were extracted from an electronic medical record system. Multivariate logistic regression was conducted to investigate factors associated with prescribing SGLT2is.

**Results:**

SGLT2is were prescribed for appropriate indication in 400 patients (100%), while inappropriately prescribed in relation to contraindication and dosing in 14 patients (3.5%). Male patients were more likely to be prescribed SGLT2is (odds ratio [OR], 1.69; 95% confidence interval [CI], 1.02–2.82). Patients with a baseline glycated hemoglobin (HbA1c) above 7% and atherosclerotic cardiovascular disease (ASCVD) were more likely to be prescribed SGLT2is (OR, 3.22; 95% CI, 1.84–5.64) and (OR, 2.18; 95% CI, 1.05–4.52), respectively. Patients receiving metformin (OR, 7.56; 95% CI, 4.46–12.80), sulfonylureas (OR, 2.30; 95% CI, 1.16–4.56), and dipeptidyl peptidase 4 inhibitors (OR, 3.43; 95% CI, 2.00–5.87) were more likely to be prescribed SGLT2is.

**Conclusion:**

SGLT2is were found to be typically prescribed for the appropriate indication. Among the most important factors associated with prescribing SGLT2is are having uncontrolled HbA1c, history of ASCVD, and using other ADDs.

## Impact statements

- The study provided new information regarding the appropriate usage of sodium-glucose cotransporter 2 inhibitors (SGLT2is) class based on indication, dosing, and contraindication, as well as the predictors for initiating this class of drugs.
- In Qatar, clinicians are up-to-date and frequently follow updates in clinical practice with respect to prescribing SGLT2is.
- Determining the factors associated with prescribing SGLT2is can aid in policy development to foster prescribing decision of prescribers.

## Introduction

Diabetes mellitus (DM) has been rising in prevalence at an alarming rate, posing a significant public health concern globally [1]. According to the International Diabetes Federation, 537 million adults (20–79 years) were diagnosed with DM in 2021 [2]. Furthermore, it is anticipated that more than 640 million adults will have DM by 2030 [2]. Patients with DM are at increased risk of cardiovascular disease (CVD)- and renal disease-related morbidity and mortality [3, 4]. Consequently, clinical practice guidelines for diabetes management have shifted their priority from achieving glycemic control alone to include the prevention and management of other comorbid health conditions [5–10].

Sodium-glucose co-transporter 2 inhibitors (SGLT2is) are the most recently approved class of oral antidiabetic drugs (ADDs). These agents work by blocking glucose reabsorption in the proximal renal tubules, thereby promoting glucose excretion in the urine and decreasing the levels of blood glucose [11]. However, SGLT2is have been associated with an increased risk of bone fractures [12], urinary tract infections (UTIs) [13], metabolic acidosis [14], and amputations [15]. Recent findings from renal and cardiovascular outcome trials have demonstrated that this class of ADDs has both cardio- and renal-protective effects [16–26]. Accordingly, several clinical practice guidelines have recommended SGLT2is as appropriate initial therapy for patients with type 2 diabetes mellitus (T2DM) with or at high-risk for atherosclerotic cardiovascular disease (ASCVD), heart failure, or chronic kidney disease (CKD) [5–10].

SGLT2is have been steadily added to many national formularies worldwide, which has subsequently led to their increased prescribing [27]. For an SGLT2i prescription to be appropriate, factors such as compelling indication and kidney function should be considered. Inappropriate prescribing has been associated with increased health care expenses and readmissions to hospitals [28, 29]. Therefore, identification of potentially inappropriate prescribing is essential in order to design the necessary interventions to improve the rational use of medications. Moreover, the decision to prescribe an SGLT2i may be influenced by several factors such as the patient's socio-demographic and clinical characteristics, physician’s experience and familiarity with the drug, previous adverse effects experience, patient preferences, medication costs, and insurance coverage [30–32].

There is paucity of information about the rational use of SGLT2is in clinical setting globally. With regards to the factors associated with prescribing SGLT2is, studies have shown that younger age [33–35], male gender [35–37], history of hypertension [36–38], history of dyslipidemia [33, 34], history of HF or coronary artery disease [33, 35, 39], overweight and obesity [35, 37] were associated with higher tendency for prescribing this class. To our knowledge, there were no previous pharmacoepidemiologic studies in the Middle East and North Africa region that evaluated the appropriateness of SGLT2is prescribing, or the factors associated with SGLT2is initiation.

### Aim

This study aimed to: (1) evaluate the appropriateness of prescribing SGLT2is based on indication, dosing, and contraindication according to American and Canadian labeling standards, and; (2) determine the factors associated with their initial prescribing.

### Ethics approval

The Institutional Review Board (IRB) at Hamad Medical Corporation (HMC) (MRC-01–20-1055) and Qatar University (1471-E/21) approved the study protocol.

## Method

### Study design and setting

This was a cross-sectional study aiming to determine the appropriateness of SGLT2is (dapagliflozin 10 mg, and empagliflozin 10 mg and 25 mg) prescribing based on indication, dosing, and contraindication according to the American and Canadian labeling standards, and the factors associated with their prescribing when compared to other oral ADDs (metformin, sulfonylureas [SUs], dipeptidyl peptidase 4 inhibitors [DPP4is], thiazolidinediones [TZDs], meglitinides [MEGs], and alfa-glucosidase inhibitors [AGIs]). This study was carried out at HMC, in Doha, Qatar. HMC is the dominant public healthcare provider of secondary and tertiary healthcare in the State of Qatar. More than 80% of the population in Qatar receive medical services through HMC facilities [40]. Information in the drug labels from the United States (U.S.) and Canada were used due to the unavailability of a validated local prescribing information or user guide for SGLT2is at HMC. In addition, clinicians at HMC use Lexicomp which already contains U.S. and Canadian labeling information.

### Study population

The study population comprised of all patients diagnosed with T2DM, who were prescribed any oral ADDs as in- or out-patients during the year 2020. The study’s inclusion criteria included: (1) adult patients (≥ 18 years old); (2) patients newly prescribed dapagliflozin between June 2020 and December 2020, or empagliflozin between May 2020 and December 2020, and/or any oral ADDs at any time during 2020; (3) patients who received the relevant medications for at least 3 days. Patients newly initiated on dapagliflozin from June—December 2020 or empagliflozin from May—December 2020 were included in the analysis. This period was selected because the U.S. Food and Drug Administration (FDA) approved dapagliflozin for reducing the risk of cardiovascular (CV) death and hospitalization for heart failure (HHF) in patients with heart failure with reduced ejection fraction (HF*r*EF) in May 2020 [41]. Meanwhile, the Canadian drug label of empagliflozin was expanded in April 2020 to include decreasing the incidence of CV mortality in patients with T2DM and established CVD [42]. Patients < 18 years old, and those who were prescribed SGLT2is or other oral ADDs for a duration of less than 3 days were excluded from the study.

### Sample size and sampling technique

The sample size for evaluating the appropriateness of prescribing SGLT2is was calculated based on the following formula:

$${\text{n}} = \frac{{Z^{2} P\left( {1 - P} \right)}}{{d^{2} }}~[43]$$

Given an expected prevalence (P) of 50%, and a precision (d) of 0.05, and a Z value of 1.96, the sample size (n) was calculated as 384. Due to the lack of data related to the expected prevalence of the appropriateness of prescribing SGLT2is in the literature, we assumed that the prevalence was going to be between 10 and 90%. Therefore, based on this assumption, it was appropriate to use a precision of 5%, and a prevalence of 50% as this value would yield a larger sample size [44, 45]. After increasing the sample size by 5% to account for missing data, the sample size was determined to be 400 subjects newly initiated on SGLT2is (200 on dapagliflozin and 200 on empagliflozin). All subjects with full information on SGLT2is prescriptions were sequentially screened for new initiators starting from June—December 2020 for dapagliflozin and from May—December 2020 for empagliflozin until 200 subjects were obtained in each group.

For determining the factors associated with prescribing SGLT2is compared to other oral ADDs, the sample size was calculated according to Hosmer, Lemeshow, and Sturdivant (2013) approach which recommends 20 observations per independent variable in the model [46]. Therefore, with a total of 29 variables identified, the sample size was calculated as 580 patients. To account for missing data, the sample size was increased by approximately 10% to become 650 (400 patients in SGLT2is initiators group and 250 in other ADDs initiators group). The 250 patients newly started on any oral ADDs were evenly divided into six groups of ADD classes (metformin, SUs, DPP4is, TZDs, MEGs, and AGIs). Prescriptions of oral ADDs were sequentially screened for new initiators starting from June—December 2020 until 250 patients were obtained, and their data were collected and included in the analysis. Therefore, a convenience sampling technique was used for selection of subjects into the study.

### Data collection instrument and procedures

Two predesigned data collection forms were developed to extract data from the HMC’s electronic medical record system (Cerner®). Information on all the prescriptions of SGLT2is and other oral ADDs during the specified periods in 2020 were extracted from Cerner® using two Excel sheets, and were screened for new initiation. Thereafter, any patient newly started on dapagliflozin between June 2020 and December 2020, empagliflozin between May 2020 and December 2020, or other oral ADDs between June and December 2020 were included for further data collection from Cerner®. The data were collected from Cerner® between 1 June 2022 and 31 August 2022.

After the data were collected, duplicates were removed based on the patient’s medical record number. Thereafter, the data were imported from Excel into Statistical Package for Social Sciences (SPSS®) version 28 (IBM Corp, Armonk, New York, USA) for analyses.

### Outcome measures

The primary outcome measure included the prevalence of inappropriate prescribing of SGLT2is (dapagliflozin and empagliflozin) based on indication, dosage, and contraindication according to American and the Canadian labelling standards (Table 1) [41, 42, 47, 48]. The secondary outcome measure comprised the factors associated with prescribing SGLT2is compared to other oral ADDs.

**Table 1:** Dapagliflozin and empagliflozin indications, dosing, and contraindication based on the American and the Canadian labeling standards

	U.S. labeling standards	Canadian labeling standards
**Dapagliflozin**		
Normal renal function		
T2DM		
• To improve glycemic control	The initial dose is 5 mg once daily. The dose can be increased to 10 mg once daily in patients tolerating 5 mg for additional glycemic control 10 mg once daily	The initial dose is 5 mg once daily. The dose can be increased to 10 mg once daily in patients tolerating 5 mg for additional glycemic control
• To reduce the risk of HHF in adults with T2DM and established CVD or multiple CV risk factors		
HF*r*EF	10 mg once daily	
Renal impairment		
*T2DM*		
• To improve glycemic control	CrCl ≥ 45 mL/min: No dosage adjustment CrCl = 30 to < 45 mL/min: Not recommended CrCl < 30 mL/min: Contraindicated ESRD/Dialysis: Contraindicated	CrCl ≥ 60 mL/min: No dosage adjustment CrCl < 60 mL/min: Contraindicated ESRD/Dialysis: Contraindicated
• To reduce the risk of HHF in adults with T2DM and established CVD or multiple CV risk factors	CrCl ≥ 45 mL/min: No dosage adjustment CrCl = 30 to < 45 mL/min: Insufficient data to support a dosing recommendation CrCl < 30 mL/min: Insufficient data to support a dosing recommendation ESRD/Dialysis: Contraindicated	
HF*r*EF	CrCl ≥ 45 mL/min: No dosage adjustment CrCl = 30 to < 45 mL/min: No dosage adjustment CrCl < 30 mL/min: Insufficient data to support a dosing recommendation ESRD/Dialysis: Contraindicated	
**Empagliflozin**		
Normal renal function		
T2DM		
• To improve glycemic control • To reduce the risk of CV death in adults with T2DM and established CVD	10 mg once daily. The dose can be increased to 25 mg once daily in patients tolerating 10 mg	10 mg once daily. The dose can be increased to 25 mg once daily in patients tolerating 10 mg
Renal impairment		
T2DM		
• To improve glycemic control • To reduce the risk of CV death in adults with T2DM and established CVD	CrCl ≥ 45 mL/min: No dosage adjustment CrCl = 30 to < 45 mL/min: Not recommended CrCl < 30 mL/min: Contraindicated ESRD/Dialysis: Contraindicated	CrCl ≥ 45 mL/min: No dosage adjustment CrCl = 30 to < 45 mL/min: Not recommended CrCl < 30 mL/min: Contraindicated ESRD/Dialysis: Contraindicated
Contraindications	History of hypersensitivity reaction to empagliflozin Severe kidney dysfunction, ESRD, or dialysis	History of hypersensitivity reaction to empagliflozin CrCl < 30 mL/min, ESRD, or dialysis

CrCl, creatinine clearance; CV, cardiovascular; CVD, cardiovascular disease; ESRD, end stage renal disease; HHF, hospitalization for heart failure; HF*r*EF, heart failure with reduced ejection fraction; T2DM, type 2 diabetes mellitus; U.S., United States

### Data analyses

Both descriptive and inferential statistical analyses were performed using SPSS version 28 (IBM Corp, Armonk, New York, USA). Continuous variables were expressed using median (interquartile range [IQR]), while categorical variables were expressed using frequencies and percentages. Baseline characteristics were compared using either Mann–Whitney U test for continuous variables or Chi-square-Goodness of Fit test for categorical variables.

Inappropriateness of prescribing dapagliflozin and empagliflozin was compared using Chi-square-Goodness of Fit test. Univariate binary logistic regression analysis was performed to determine the factors associated with prescribing SGLT2is compared to other oral ADDs. Factors that showed significant association with prescribing SGLT2is (i.e. with *p* values < 0.25) in the univariate analysis were incorporated into the multivariate binary logistic regression model for further analysis. *P*-value less than 0.05 was considered statistically significant in the multivariate binary logistic regression model.

## Results

### Characteristics of the study subjects

Six hundred and fifty patients with T2DM were included in the study, with 400 (61.5%) in the SGLT2is group with 200 (30.8%) in the dapagliflozin and 200 (30.8%) in the empagliflozin groups, and 250 (38.5%) in the other ADDs group (Table 2). The median (IQR) baseline glycosylated hemoglobin A1c (HbA1c) of the patients in the SGLT2is group was significantly higher than that of patients in the other oral ADDs group [9% (2.8) vs. 7% (2), (*p* < 0.001)]. Furthermore, there was a significantly greater proportion of patients with CrCl < 60 mL/min/1.73m^2^ in the other oral ADDs group compared to the SGLT2is group, (*p* < 0.001). Hypertension was the most common comorbidity in both groups [53.0% vs. 70.0%, (*p* < 0.001)], followed by dyslipidemia [47.5% vs. 45.2%, (*p* = 0.567)].

**Table 2:** Baseline characteristics of patients initiated on sodium-glucose co-transporter 2 inhibitors and other oral antidiabetic drugs during 2020

Variable	SGLT2is vs. other oral ADDs			Dapagliflozin vs. Empagliflozin		
	SGLT2is (n = 400)	Oral ADDs (n = 250)	*P* value^a,b^	Dapagliflozin (n = 200)	Empagliflozin (n = 200)	*P* value^a,b^
Age (years), median (IQR)	52 (17)	59 (17)	< 0.001^a^	50 (17)	54 (16)	0.001^a^
Age category, n (%)			< 0.001^b^			0.438^b^
< 65	353 (88.3)	165 (66.0)		179 (89.5)	174 (87.0)	
≥ 65	47 (11.8)	85 (34.0)		21 (10.5)	26 (13.0)	
Gender, n (%)			0.005^b^			0.282^b^
Male	274 (68.5)	114 (57.6)		132 (66.0)	142 (71.0)	
Female	126 (31.5)	106 (42.4)		68 (34.0)	58 (29.0)	
Nationality, n (%)			0.125^b^			0.483^b^
Arabs	209 (52.3)	146 (58.4)		101 (50.5)	108 (54.0)	
Non-Arabs	191 (47.8)	104 (41.6)		99 (49.5)	92 (46.0)	
BMI (Kg/m^2^), median (IQR)	30 (8)	29 (8)	0.561^a^	30 (8)	29 (8)	0.404^a^
BMI category (Kg/m^2^), n (%) *			0.877^b^			0.091^b^
Normal (18.5–24.9)	62 (16.2)	44 (17.7)		31 (16.1)	31 (16.3)	
Overweight (25–29.9)	128 (33.4)	81 (32.7)		55 (28.5)	73 (38.4)	
Obese (≥ 30)	193 (50.4)	123 (49.6)		107 (55.4)	86 (45.3)	
Baseline HbA1c, median (IQR)^c^	9 (2.8)	7 (2)	< 0.001^a^	9.2 (3.0)	8.8 (2.8)	0.352^a^
Baseline HbA1c category, n (%) *			< 0.001^b^			0.465^b^
HbA1c ≤ 7%	55 (14.3)	124 (50.8)		28 (14.4)	27 (14.3)	
HbA1c 7.1–8.9%	127 (33.1)	73 (29.9)		59 (30.3)	68 (36.0)	
HbA1c ≥ 9%	202 (52.6)	47 (19.3)		108 (55.4)	94 (49.7)	
Latest HbA1c, median (IQR)^d^	7.70 (2.2)	7.05 (2.1)	< 0.001^a^	7.7 (2.2)	7.6 (2.2)	0.839^a^
Latest HbA1c category, n (%) *			< 0.001^b^			0.525^b^
HbA1c ≤ 7%	120 (31.3)	114 (50.0)		57 (29.7)	63 (32.8)	
HbA1c 7.1–8.9%	163 (42.4)	70 (30.7)		87 (45.3)	76 (39.6)	
HbA1c ≥ 9	101 (26.3)	44 (19.3)		48 (25.0)	53 (27.6)	
CrCl category, n (%) *			< 0.001^b^			0.693^b^
CrCl ≥ 60	359 (89.8)	164 (67.8)		182 (91.0)	177 (88.5)	
CrCl 45–59	27 (6.8%)	30 (12.0)		12 (6.0)	15 (7.5)	
CrCl 30–44	13 (3.3%)	33 (13.2)		6 (3.0)	7 (3.5)	
CrCl < 30	1 (0.3)	18 (7.2)		0 (0)	1 (0.5)	
Comorbidities, n (%)						
Hypertension	212 (53)	175 (70.0)	< 0.001^b^	100 (50.0)	112 (56.0)	0.229^b^
Dyslipidemia	190 (47.5)	113 (45.2)	0.567^b^	102 (51.0)	88 (44.0)	0.161^b^
AHF*r*EF	15 (3.8)	8 (3.2)	0.712^b^	6 (3.0)	9 (4.5)	0.430^b^
SCVD	86 (21.5%)	35 (14.0)	0.017^b^	22 (11.0)	64 (32.0)	< 0.00^b^
CKD	25 (6.3)	58 (23.2)	< 0.00^b^	12 (6.0)	13 (6.5)	0.836^b^
Others	81 (20.3)	80 (32.0)	< 0.001^b^	31 (15.5)	50 (25.0)	0.018^b^
History of UTIs, n (%)	5 (1.3)	10 (4.0)	0.023^b^	4 (2.0)	1 (0.5)	0.177^b^
History of bone fractures, n (%)	2 (0.5)	9 (3.6)	0.004^b^	2 (1.0)	0 (0)	0.156^b^
Concomitant ADDs, n (%)						
Metformin	353 (88.3)	69 (27.6)	< 0.001^b^	179 (89.5)	174 (87.0)	0.438^b^
SUs	130 (32.5)	21 (8.4)	< 0.001^b^	69 (34.5)	61 (30.5)	0.393^b^
DPP4is	272 (68.0)	44 (17.6)	< 0.001^b^	147 (73.5)	125 (62.5)	0.018^b^
TZDs	18 (4.5)	4 (1.6)	0.047^b^	12 (6.0)	6 (3.0)	0.148^b^
GLP-1 RAs	9 (2.3)	11 (4.4)	0.123^b^	4 (2.0)	5 (2.5)	0.736^b^
Insulin	118 (29.5)	82 (32.8)	0.375^b^	54 (27.0)	64 (32.0)	0.273^b^
MEGs	5 (1.3)	3 (1.2)	0.955^b^	4 (2.0)	1 (0.5)	0.177^b^
AGIs	2 (0.5)	0 (0)	0.263^b^	2 (1.0)	0 (0)	0.156^b^
Other concomitant medications, n (%)						
Aspirin	119 (29.8)	84 (33.6)	0.303^b^	37 (18.5)	82 (41.0)	< 0.001^b^
RAASis	193 (48.3)	132 (52.8)	0.259^b^	80 (40.0)	113 (56.5)	< 0.001^b^
Sacubitril/valsartan	10 (2.5%)	1 (0.4%)	0.043^b^	4 (2.0)	6 (3.0)	0.522^b^
BBs	98 (24.5)	68 (27.2)	0.443^b^	31 (15.5)	67 (33.5)	< 0.001^b^
CCBs	85 (21.3)	81 (32.4)	0.002^b^	44 (22.0)	41 (20.5)	0.714^b^
Diuretics	59 (14.8)	66 (26.4	< 0.001^b^	22 (11.0)	37 (18.5)	0.034^b^
Statins	247 (61.8)	148 (59.2)	0.517^b^	114 (57.0)	133 (66.5)	0.051^b^
Others	222 (55.5)	190 (76.0)	< 0.001^b^	104 (52.0)	118 (59.0)	0.159^b^

ADD, antidiabetic drug; AGI, alpha-glucosidase inhibitor; ASCVD, atherosclerotic cardiovascular disease; BB, beta blocker; BMI, body mass index; CCBs, calcium channel blockers; CKD, chronic kidney disease; CrCl, creatinine clearance; DPP4i, dipeptidyl peptidase 4 inhibitor; GLP-1 RA, glucagon-like peptide-1 receptor agonist; HbA1c, glycated hemoglobin; HF*r*EF, heart failure with reduced ejection fraction; IQR, interquartile range; MEG, meglitinide; RAASi, renin–angiotensin–aldosterone system inhibitor; SU, sulfonylurea; TZD, thiazolidinedione; UTI, urinary tract infection

^*^Missing data

^a^Mann–Whitney U test and

^b^Chi-square-Goodness of Fit test were used to compute the *p*-values

^c^Baseline HbA1c level represents the most recent HbA1c reading preceding the prescribing of oral ADDs or SGLT2i

^d^Latest HbA1c level represents the latest HbA1c reading in 2020, which was 3–6 months post- oral ADDs or SGLT2i prescribing

### Appropriateness of prescribing sodium-glucose co-transporter 2 inhibitors

All 400 patients in the SGLT2i group (100%) that were included in this study were prescribed SGLT2is for appropriate indications, and had T2DM as the primary indication. Dapagliflozin was prescribed for: improving glycemic control in patients with T2DM for 125 patients (62.5%), HFrEF for six patients (3%), risk reduction of HHF in patients with T2DM and established CVD for 22 patients (11%), and risk reduction of HHF in adults with T2DM and multiple CV risk factors for 47 patients (23.5%). On the other hand, empagliflozin was prescribed for: improving glycemic control in patients with T2DM for 136 patients (68%) and the risk reduction of CV death in adults with T2DM and established CVD for 64 patients (32%).

SGLT2is were inappropriately prescribed in relation to contraindication and dosing in 14 patients (3.5%) based on the renal function. Thirteen had CrCl level ranging from 31 to 41 mL/min, while one patient had CrCl < 30 mL/min. When comparing prescribing inappropriateness of dapagliflozin versus empagliflozin, there was no statistically significant difference between the two groups [2.5% vs. 4.5%, (*p* = 0.31)].

### Factors associated with prescribing sodium-glucose co-transporter 2 inhibitors

In the multivariate logistic regression analysis, males were more likely to be prescribed SGLT2is than females with an odds ratio (OR) of 1.69 (95% confidence interval (CI), 1.02 to 2.82; *p* = 0.044). Compared to patients with baseline HbA1c of less than 7%, SGLT2is were more likely to be prescribed among those with a baseline HbA1c of more than 7% (OR, 3.22; 95% CI, 1.84 to 5.64; *p* < 0.001). Furthermore, SGLT2is were more likely to be prescribed among those with ASCVD compared to patients with no history of ASCVD (OR, 2.18; 95% CI, 1.05 to 4.52; *p* = 0.036). In contrast, SGLT2is were less likely to be prescribed among patients with history of CKD compared to those with no CKD (OR, 0.36; 95% CI, 0.15–0.87; *p* = 0.024).

In terms of the concurrent ADDs, patients on metformin, SUs, or DPP4is were more likely to be prescribed SGLT2is than patients not on those ADDs with ORs of 7.56 (95% CI, 4.46 to 12.80; *p* < 0.001), 2.30 (95% CI, 1.16 to 4.56; *p* = 0.017), and 3.43 (95% CI, 2.00 to 5.87; *p* < 0.001), respectively. There was no evidence for differences in prescribing SGLT2is by ethnicity, CrCl level (< 60 or ≥ 60 mL/min), history of urinary tract infections, history of bone fractures, and concurrent administration of TZDs, sacubitril/valsartan, or diuretics (Table 3).

**Table 3:** Multivariate binary logistic regression of the factors associated with prescribing sodium-glucose co-transporter 2 inhibitors compared to other oral antidiabetic drugs

Variable	Adjusted OR	95% CI	*P*-value
Age category (≥ 65 vs. < 65)	0.657	0.337–1.281	0.218^a^
Gender (Male vs. Female)	1.692	1.015–2.822	0.044^a^
Ethnicity category (Non-Arabs vs. Arabs)	0.976	0.591–1.611	0.923^a^
Baseline HbA1c category (> 7 vs. ≤ 7)	3.219	1.838–5.637	< 0.001^a^
CrCl category (< 60 vs. ≥ 60)	0.978	0.438–2.180	0.956^a^
Hypertension	0.576	0.318–1.043	0.069^a^
ASCVD	2.182	1.053–4.523	0.036^a^
CKD	0.359	0.148–0.872	0.024^a^
History of UTIs	0.896	0.180–4.474	0.894^a^
History of bone fractures	0.000	0.000	0.998^a^
Metformin	7.556	4.460–12.802	< 0.001^a^
SUs	2.301	1.160–4.563	0.017^a^
DPP4is	3.430	2.004–5.871	< 0.001^a^
TZDs	0.907	0.230–3.578	0.890^a^
GLP-1 RAs	1.602	0.487–5.264	0.438^a^
Sacubitril/valsartan	6.385	0.812–50.182	0.078^a^
CCBs	1.381	0.748–2.550	0.302^a^
Diuretics	0.811	0.422–1.560	0.531^a^

ASCVD, atherosclerotic cardiovascular disease; CCB, calcium channel blocker; CKD, chronic kidney disease; CI, confidence interval; CrCl, creatinine clearance; DPP4i, dipeptidyl peptidase 4 inhibitor; GLP-1 RA, glucagon-like peptide-1 receptor agonist; HbA1c, glycated hemoglobin; OR, odds ratio; SU, sulfonylurea; TZD, thiazolidinedione; UTI, urinary tract infection

^a^Multivariate binary logistic regression test was used to compute the *p*-values

Nagelkerke R^2^ = 0.602

## Discussion

### Statement of key findings

This study demonstrated that in the studied cohort, SGLT2is were frequently prescribed appropriately in relation to contraindication and dosing, and were always prescribed appropriately in relation to indication according to international labeling standards [41, 42, 47, 48]. Gender, baseline HbA1c, history of ASCVD, and the use of metformin, SUs, and DPP4is had a positive influence on prescribing SGLT2is compared to other oral ADDs. Meanwhile, history of CKD had a negative influence on prescribing SGLT2is.

### Interpretation

Appropriate prescribing of SGLT2is in relation to indication and contraindication indicates that physicians in our study adhere to internationally-recognized labeling standards. Despite the U.S. FDA approval of dapagliflozin for reducing the risk of CV mortality and HHF in patients with HF*r*EF, the proportion of patients who were prescribed dapagliflozin for this indication was lower than that in the empagliflozin group. Similarly, the proportion of patients prescribed an SGLT2i for the indication of reducing the risk of CV death in patients with T2DM and established ASCVD was significantly higher in the empagliflozin group compared to dapagliflozin group. This may be attributed to the fact that the drug label of empagliflozin for this indication was expanded by the U.S. FDA in 2016 and the Canadian drug labelling in 2020 [42, 49].

Patients were more likely to be prescribed a SGLT2i if they had a baseline HbA1c > 7%. This is in accordance with clinical practice guidelines which generally recommend additional and/or alternative agents to achieve glycemic control following the failure of metformin monotherapy, as well as considering patient-centered treatment factors [5–10]. Male gender was associated with higher odds of prescribing SGLT2is, which is similar to previous studies conducted in the US and UK [35, 37]. Moreover, using SGLT2is is associated with a higher risk of UTIs [13], and female patients are more likely to develop UTIs compared to male patients [50]. Regarding the influence of comorbidities on SGLT2is initiation, the presence of ASCVD appears to be a key driver for SGLT2is prescribing. A study conducted in the US demonstrated that coronary artery disease (CAD) was associated with a higher probability of SGLT2is initiation [35]. Given the observed CV benefits of SGLT2is in the EMPA-REG OUTCOME trial, published in 2015 [16], clinical practice guidelines recommended special consideration of this class in the context of CVD since 2016 [51–53]. Our study indicates that physicians prescribe SGLT2is using evidence from landmark cardiovascular outcome trials and evidence-based guidelines.

The findings of the present study suggest that patients with a history of CKD were less likely to be prescribed SGLT2is. This is in spite of the publication of dedicated kidney outcome trials including CREDENCE and DAPA-CKD trials [19, 22], as well as the updated guidelines recommendations promoting the use of this novel class in patients with DM and CKD since 2019 [54, 55]. Nonetheless, it is worth noting that a study in the US showed that the presence of CKD significantly decreased the likelihood of prescribing SGLT2is [35]. Patients with CKD usually have multiple comorbid conditions, which might lead physicians to be more hesitant to initiate a new medication, either because of concerns related to adverse effects or existing polypharmacy. We anticipate that the utilization of SGLT2is among patients with CKD may increase over time as the evidence supporting their benefits continue to emerge. The influence of other ADDs on prescribing SGLT2is was noticed, whereby patients on metformin, SUs, or DPP4is were more likely to be prescribed SGLT2is. This is in line with studies conducted in other countries [33, 36, 38].

### Strengths and weaknesses

This study contributes to the existing body of knowledge and practice through providing new information regarding the appropriate usage of SGLT2is class based on indication, dosing, and contraindication, as well as the predictors for initiating this class of drugs. The findings can reflect how clinicians in our setting are up-to-date and apply the recent updates in clinical practice. On the other hand, this study has some limitations. First, no data were available from community pharmacies and private hospitals as it was difficult to obtain an estimate about the percentage of data from the private sector. However, the study was conducted in the medical sector which provides healthcare services to more than 80% of the population in Qatar [40]. Second, the appropriateness of prescribing SGLT2is was not evaluated according to the European labeling standards as they were not available at the European Medicines Agency (EMA) website. Third, the factors associated with prescribing SGLT2is should be optimally evaluated at three levels including patient-related, prescriber-related, and system-related. However, it was not feasible to capture such data from Cerner®. Despite our efforts to account for most of factors, other factors could be potentially missed due to the retrospective nature of the study. Finally, cross-sectional studies provide data that are only valid at the time of collection [56].

### Further research

Factors associated with initiating SGLT2is are multifactorial in nature and our study focused on patient-related factors. Therefore, future studies should investigate the factors at the prescriber and system-level, as well as the potential barriers for prescribing SGLT2is. Following the publication of studies that demonstrated the benefits of SGLT2is in patients with CKD, HF*r*EF, and HF with mildly reduced or persevered ejection fraction, regardless of the presence or absence of DM, from the end of 2020 until the end of 2022 [22–25], investigating the adoption and application of their results in practice may be an interesting area for future studies.

## Conclusion

This study provides additional information to the existing literature regarding the appropriateness of prescribing SGLT2is and the factors influencing their prescribing. SGLT2is were found to be typically prescribed for the appropriate indication. Among the most important factors associated with prescribing SGLT2is are having uncontrolled HbA1c, history of ASCVD, and using other oral ADDs.
